# Genome-Wide Identification and Expression Profiling of the RNA-Directed DNA Methylation Pathway Genes in *Cucumis sativus* L.

**DOI:** 10.3390/plants14182908

**Published:** 2025-09-18

**Authors:** Li Ma, Ziyi Li, Lei Qiu, Jieni Gu, Piaopiao Shi, Xinyi Cao, Xinran Zhang, Xi Xu, Yinbo Ma

**Affiliations:** College of Horticulture and Landscape Architecture, Yangzhou University, Yangzhou 225009, China; mali040929@gmail.com (L.M.);

**Keywords:** RNA-directed DNA methylation (RdDM), salinity, drought, heavy metals, cucumber

## Abstract

The RNA-directed DNA methylation (RdDM) pathway is a crucial epigenetic mechanism governing plant responses to environmental stress. While the RdDM pathway has been extensively studied in *Arabidopsis thaliana*, the comprehensive understanding of its components in cucumber (*Cucumis sativus* L.) remains lacking. In this study, we performed a genome-wide identification and characterization of RdDM pathway genes in cucumber, followed by an analysis of their expression patterns across various tissues and under multiple abiotic stress conditions. A total of 67 putative *CsRdDM* genes were identified, which are unevenly distributed across the cucumber’s chromosomes. Phylogenetic and gene structure analyses revealed considerable evolutionary divergence, particularly within the key Argonaute gene family (*CsAGO*). Crucially, the promoter regions of *CsRdDM* genes were found to contain cis-regulatory elements associated with abiotic stress, light signaling, and development, suggesting their potential involvement in complex regulatory networks. RT-qPCR assays confirmed that *CsRdDM* genes exhibit distinct and stress-specific transcriptional patterns. Notably, several genes such as *CsAGO4* and *CsIDN2* showed antagonistic expression between roots and leaves under drought (PEG-6000) stress, implying a sophisticated, tissue-specific defense mechanism. Among them, *CsAGO4* emerged as a candidate gene responsive to abiotic stress. Those findings provide new insights into the regulatory roles of *CsRdDM* genes under abiotic stress and highlight candidate genes for the genetic improvement of stress tolerance in cucumber.

## 1. Introduction

Cucumber (*Cucumis sativus* L.) is a widely cultivated vegetable crop of substantial nutritional and economic importance, serving as a rich source of vitamins and minerals in the human diet [1,2,3]. However, cucumber productivity and quality are often compromised by various environmental challenges, including abiotic stresses such as heavy metal contamination [4], drought [5], and high salinity [6], which lead to significant agricultural losses. Therefore, developing stress-tolerant cucumber cultivars through modern biotechnological and molecular breeding approaches is essential for achieving sustainable and efficient crop production [7]. A critical step in this process is elucidating the regulatory mechanisms by which plants perceive and respond to environmental stress. Among these mechanisms, epigenetic regulation has emerged as a central component of plant adaptability to adverse conditions [8].

Epigenetic modifications, including DNA methylation, histone modifications, and chromatin remodeling, offer a flexible and reversible means of gene regulation that enables plants to modulate their phenotypes in response to environmental cues without altering their genetic code [9,10,11]. Among these, the RNA-directed DNA methylation (RdDM) pathway is a plant-specific, highly conserved mechanism that plays a pivotal role in transcriptional gene silencing, maintenance of genome integrity, and stress response regulation [12]. This pathway is mediated by a specialized set of components, including two plant-specific RNA polymerases (Pol IV and Pol V) and a core suite of regulatory proteins [13]. Pol IV is recruited to target genomic regions, primarily transposons and repetitive sequences, where it transcribes single-stranded RNAs. These transcripts are converted into double-stranded RNAs by RNA-dependent RNA polymerase 2. The double-stranded RNAs are then processed into 24-nucleotide small interfering RNAs by DICER-LIKE 3, and these small RNAs are stabilized through methylation at their 3′ ends by HUA ENHANCER 1 before being loaded onto ARGONAUTE proteins. Concurrently, Pol V transcribes non-coding scaffold RNAs that base-pair with the small interfering RNA-ARGONAUTE complex. This interaction recruits DOMAINS REARRANGED METHYLTRANSFERASE 2, which mediates de novo DNA methylation at homologous genomic loci across all sequence contexts [14]. Recent studies have also identified non-canonical RdDM pathways that diverge from the canonical model by utilizing RNA polymerase II (Pol II) instead of Pol IV, generating 21–22 nt siRNAs via RDR6 and DCL2/4. These siRNAs primarily mediate the initial phase of de novo methylation at newly inserted genomic elements and may transition to the canonical RdDM pathway for maintenance of methylation patterns [12,15].

The RdDM pathway has been extensively implicated in regulating plant growth, development, and responses to abiotic and biotic stresses. In Arabidopsis, for instance, *AtNRPD2* is involved in post-heat stress recovery, while *AtHDA6* enhances heat tolerance by modulating acute transcriptional responses [16]. Loss of function mutants in *AGO4* and *NRPD2A* show changed seed germination patterns under variable maternal temperatures, indicating a role for RdDM in transgenerational environmental sensing [17]. In rice, CHH methylation mediated by RdDM in the promoter of *OsGSTZ4* enhances cadmium tolerance [18]. In tomato, suppression of *SlAGO4A* improves tolerance to salt stress and drought by modulating the methylation levels of stress response genes [19]. Similarly, Pol V mutants in Arabidopsis exhibit reduced resistance to fungal pathogens, highlighting the involvement of RdDM in biotic stress responses [20]. In terms of plant growth and development, Arabidopsis *met1* mutants exhibit delayed flowering due to reduced methylation levels at FWA sites, indicating that RdDM is associated with flowering time regulation [21]. In apples, *MdAGO4s* mediate CHH methylation of the *MdMYB1* promoter via RdDM, thereby influencing anthocyanin biosynthesis [22]. Despite these advancements, most functional investigations of RdDM components have been restricted to model species and conducted under controlled laboratory conditions. A comprehensive, genome-wide characterization of the RdDM pathway genes and their expression dynamics under diverse abiotic stresses in cucumber remains notably absent.

To address this knowledge gap, the present study performed a genome-wide identification and functional analysis of core RdDM pathway genes in cucumber. Through integrated bioinformatics and reverse transcription quantitative real-time PCR (RT-qPCR) assays, we systematically examined gene family composition, tissue-specific expression patterns, and transcriptional responses to abiotic stresses, including heavy metals (Cu^2+^ and Zn^2+^), salt (NaCl), and drought (PEG-6000). The results revealed dynamic changes in the expression of the RdDM pathway in cucumber’s response to abiotic stress and identified several stress-responsive candidate genes. These genes may serve as potential targets for molecular design in epigenetics-based breeding aimed at improving stress resilience in cucumber. These findings contribute to a better understanding of the epigenetic mechanisms underlying plant stress responses and offer a foundation for their application in crop improvement.

## 2. Results

### 2.1. Genome-Wide Identification and Phylogenetic Analysis of CsRdDMs

To comprehensively identify the components of the RdDM pathway genes in cucumber, a genome-wide search was conducted. Based on homology searches against annotated proteomes from eight plant species and subsequent confirmation by Pfam domain analysis, a total of 67 non-redundant genes, referred to as *CsRdDMs*, were identified as putatively involved in the cucumber DNA methylation machinery. These genes were classified into ten major families based on their homology to Arabidopsis orthologs, including five DNA methyltransferases (*CsMETs*/*CsDNMTs*), eight Argonaute proteins (*CsAGOs*), four Dicer-like proteins (*CsDCLs*), seven RNA-dependent RNA polymerases (*CsRDRs*), and additional regulatory essential factors such as *CsSHHs*, *CsSUVHs*, *CsRDMs*, *CsHDACs*, and *CsMORCs*. Detailed information for each identified gene, including genome IDs, coordinates (5′–3′), ORF (bp), protein length (aa), isoelectric point, molecular weight (KDa), and intron count, is provided in Supplementary Table S1.

**Figure 1:**
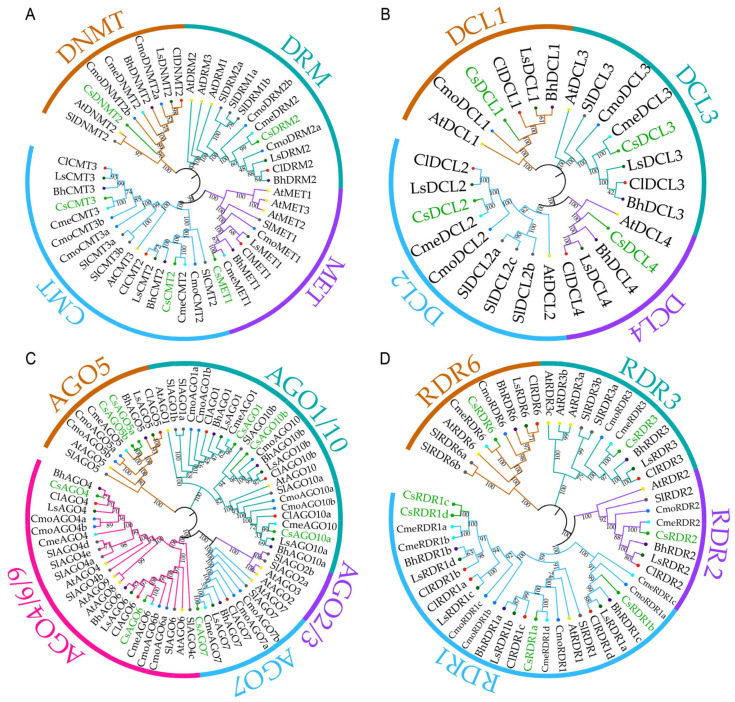
Phylogenetic analysis of the gene families in the RdDM pathway, proteins from various species, including cucumber (*Cucumis sativus*, abbreviation: Cs, 67 genes), Arabidopsis (*Arabidopsis thaliana*, abbreviation: At, 28 genes), wax gourd (*Benincasa hispida*, abbreviation: Bh, 21 genes), watermelon (*Citrullus lanatus*, abbreviation: Cl, 23 genes), pumpkin (*Cucurbita moschata*, abbreviation: Cmo, 32 genes), bottle gourd (*Lagenaria siceraria*, abbreviation: Ls, 23 genes), melon (*Cucumis melo*, abbreviation: Cme, 20 genes), and tomato (*Solanum lycopersicum*, abbreviation: Sl, 32 genes) were used in the analysis. Each node supports 1000 replicates. Phylogenetic tree of DNA methyltransferase (DNMT) proteins (**A**), Dicer-like (DCL) proteins (**B**), Argonaute (AGO) proteins (**C**), and RNA-dependent RNA polymerase (RDR) proteins (**D**). Different colors of branches in the same phylogenetic tree represent different subgroups.

To explore the evolutionary relationships of these gene families, a phylogenetic analysis was performed using homologous protein sequences from cucumber and seven other representative plant species (Figure 1). The phylogenetic trees revealed clear evolutionary patterns among the core RdDM family members. The DNMT family members were classified into four subfamilies (DNMT, DRM, MET, CMT), with the branch distribution of *Cucumis sativus* (Cs) proteins showed significant divergence from that of *A. thaliana* (At) and *Solanum lycopersicum* (Sl), suggesting potential structural or quantitative evolutionary shifts in cucumber (Figure 1A). By contrast, the four CsDCL proteins were distributed evenly into each of the four DCL subfamilies (DCL1-4), indicating a highly conserved evolutionary history for this family (Figure 1B). For the AGO family, proteins from cucurbitaceous species formed distinct clades separate from those of *Solanaceae* and *Brassicaceae*, highlighting inter-family evolutionary divergence (Figure 1C). The CsRDR family exhibited complex evolutionary patterns, with members distributed across the RDR1, RDR2, RDR3, and RDR6 subfamilies. Particularly, the tight clustering of *CsRDR1c* and *CsRDR1d* within the RDR1 subfamily suggests a recent duplication event and potential functional redundancy (Figure 1D).

### 2.2. Chromosomal Location and Duplication of CsRdDMs

**Figure 2:**
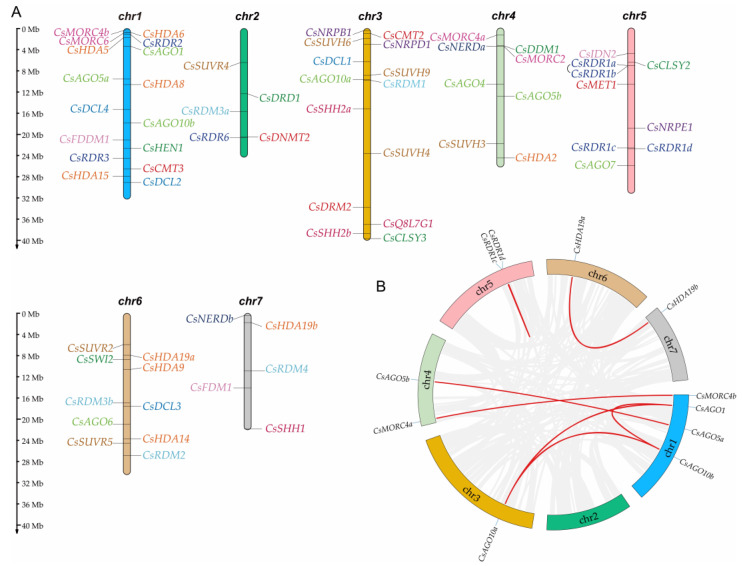
Gene location and Collinearity analysis of the RdDM genes in cucumber. (**A**) Chromosomal localization of RdDM genes in the cucumber genome. (**B**) Collinearity analysis of RdDM genes in the cucumber genome. Red lines denote collinear gene pairs among RdDM family members.

The genomic distribution of the 67 *CsRdDM* genes was mapped onto the seven cucumber chromosomes based on the “Chinese Long V3” genome (Figure 2). The genes were unevenly distributed across the chromosomes. Of particular interest, a tandem duplication event involving *CsRDR1a* and *CsRDR1b* was identified on chromosome 5. These genes encode RNA-dependent RNA polymerases that play pivotal roles in antiviral defense. Their tandem duplication may enhance the efficiency of RNA silencing and gene regulation in cucumber.

Further analysis identified seven pairs of segmental duplications involving multiple gene subfamilies. The statistics of *CsRdDM* gene sequence homology are shown in Supplementary Table S2. The *CsAGO* subfamily exhibited the most significant expansion with four duplicated pairs (*CsAGO1*/*CsAGO10a*, *CsAGO1*/*CsAGO10b*, *CsAGO10a*/*CsAGO10b*, and *CsAGO5a*/*CsAGO5b*). Single segmental duplications were also observed in the *CsMORC* (*CsMORC4a*/*CsMORC4b*), *CsRDR* (*CsRDR1c*/*CsRDR1d*), and *CsHDA* (*CsHDA19a*/*CsHDA19b*) subfamilies. The presence of multiple duplicated gene pairs in the *CsAGO* subfamily suggests a potential for functional redundancy and diversification, which would bolster the regulatory capacity and adaptive plasticity of the cucumber RdDM pathway.

### 2.3. Protein Structure Analysis of CsRdDMs

**Figure 3:**
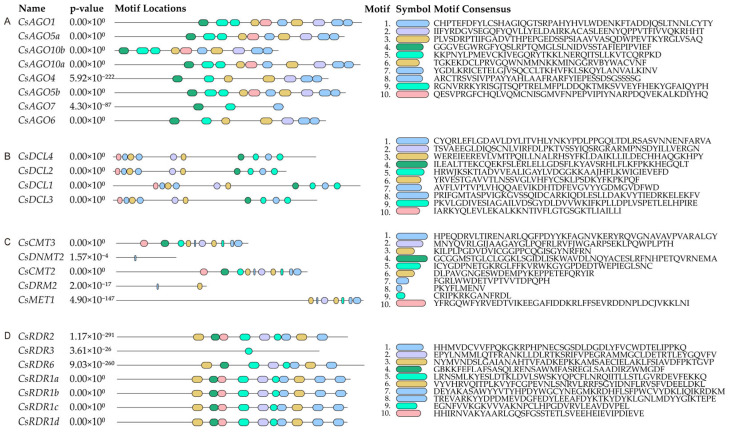
Motif analysis of the main gene families involved in the RdDM pathway. (**A**) CsAGO gene family; (**B**) CsDCL gene family; (**C**) five methyltransferase genes, including *CsCMT3*, *CsDNMT2*, *CsCMT2*, *CsDRM2*, and *CsMET1*; (**D**) CsRDR gene family.

To further investigate the evolutionary relationships among *CsRdDMs*, the conserved motifs (Figure 3) and Pfam domain (Figure S1) were analyzed. A strong correlation between phylogeny and protein structure was evident, as members within a given subfamily typically shared homologous motifs and domain patterns. It is worth noting that double strand RNA binding domain are present in both the *CsDCL1* and *CsDCL4* domains of cucumbers, while they are not detected in *CsDCL2* and *CsDCL3*. This situation is consistent with the Arabidopsis DCL family [23]. *CsAGO7* displays a reduced number of motifs compared to other members of the CsAGO family, while the remaining CsAGO members exhibit a near-identical distribution of motif types and quantities.

Within the CsRDR family, *CsRDR3* and *CsRDR6* displayed unique motif patterns compared to other members, corroborating their distinct phylogenetic positions. In contrast, the high similarity in motif distribution among *CsRDR1a*, *CsRDR1b*, *CsRDR1c*, *CsRDR1d*, and *CsRDR2* is indicative of strong evolutionary conservation within this particular clade. The domain analysis further highlighted evidence of sub-functionalization. Within the CsDCL family, for example, both *CsDCL2* and *CsDCL4* contain a core DEAD/DEAH box helicase domain (PF00270), yet *CsDCL4* possesses an additional double-stranded RNA-binding domain (PF14709). A similar structural variation between *CsDCL1* and *CsDCL3* suggests that subtle differences in domain architecture likely confer distinct functional roles upon these paralogs.

### 2.4. Gene Structure Analysis of CsRdDMs

**Figure 4:**
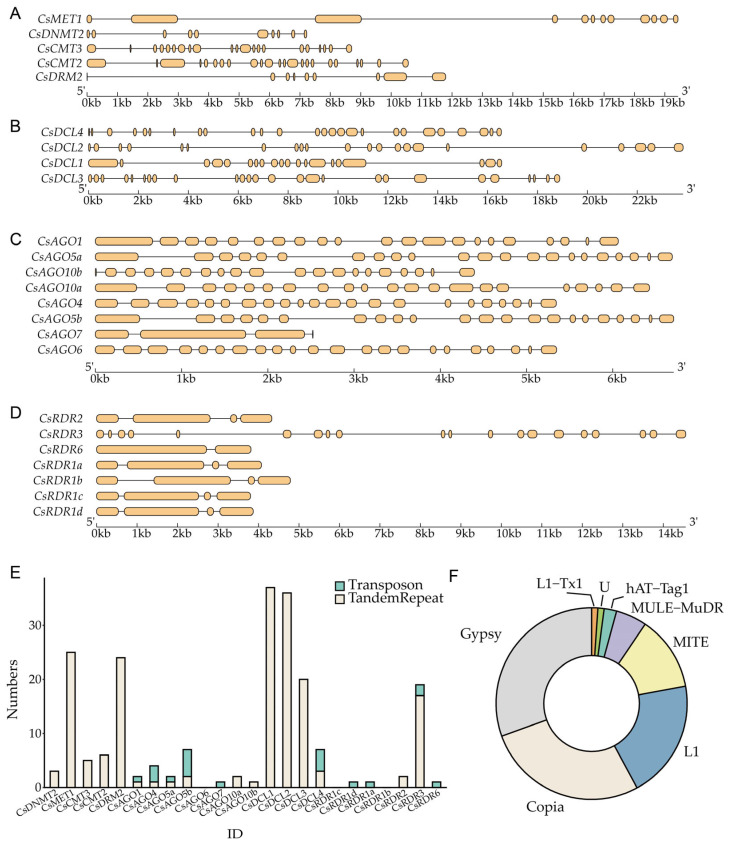
The gene structures of five methyltransferases (*CsMET1*, *CsDNMT2*, *CsCMT3*, *CsCMT2*, *CsDRM2*) and the three main gene families in the RdDM pathway (CsAGO, CsRDR, CsDCL) (**A**–**D**). (**E**) The distribution of internal repetitive sequences in five methyltransferases (*CsMET1*, *CsDNMT2*, *CsCMT2*, *CsCMT3*, *CsDRM2*), and the three main gene families in the RdDM pathway (CsAGO, CsRDR, CsDCL), where blue represents Transposons, and ochre represents Tandem Repeats. (**F**) The distribution types of internal repetitive sequences in five methyltransferases (*CsMET1*, *CsDNMT2*, *CsCMT3*, *CsCMT2*, *CsDRM2*), and the three main gene families in the RdDM pathway (CsAGO, CsRDR, CsDCL).

The exon-intron organization of genes provides critical clues into their evolutionary history [24]. Substantial variation was detected in both exon number and intron length among *CsRdDM* families (Figure 4A–D). For instance, *CsAGO7* exhibited fewer exons compared to other *CsAGO* genes, which generally harbored 20–22 exons, suggesting significant structural divergence, which is consistent with the Argonaute gene family in *Arabidopsis thaliana* and the callus of longan embryos [25]. Similarly, the distinct gene architectures of *CsRDR3* and *CsRDR6* align with their unique evolutionary paths within the CsRDR family.

Repetitive element analysis revealed differences in the distribution of transposable elements (TEs) and tandem repeats within the genomic regions of the four major *CsRdDM* families (Figure 4E). An inverse relationship was identified between tandem repeat abundance and TE density, with genes enriched in tandem repeats generally containing fewer TEs. A positive correlation was observed between total intron length and the density of repetitive sequences. Genes such as *CsMET1*, *CsDRM2*, and *CsDCL1* exhibited both long introns and a high frequency of repeats. Among the identified TEs, long terminal repeat (LTR) retrotransposons were predominant, particularly the Gypsy (28 instances) and Copia (26 instances) families, followed by LINEs (L1, 19 instances) and DNA transposons (MITE, 15 instances) (Figure 4F).

### 2.5. Cis-Regulatory Elements Analysis of CsRdDMs

**Figure 5:**
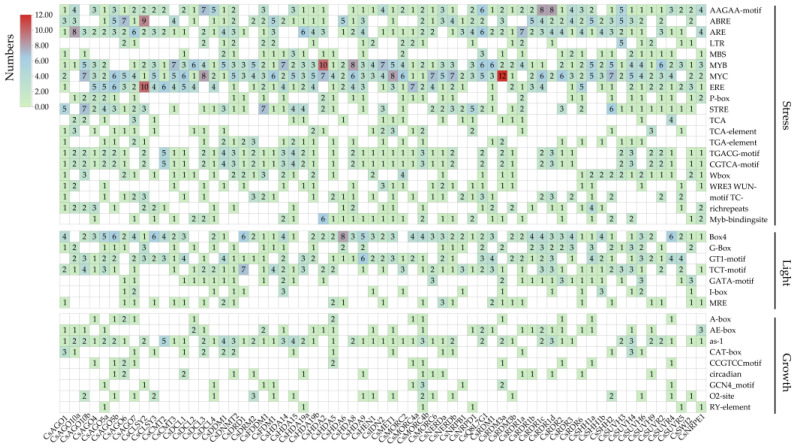
Cis-regulatory elements analysis of *CsRdDMs*. The upstream 2000 bp region of each RdDM pathway gene was used for cis-regulatory elements identification. Identification was made using the PlantCARE database. The numbers represent the count of cis-regulatory elements.

To investigate potential regulatory mechanisms of *CsRdDM* gene expression, the 2000 bp upstream promoter sequences were analyzed for cis-regulatory elements using the PlantCARE database (Figure 5). A total of 110 distinct cis-elements were identified and categorized into three major functional groups: stress-responsive, light-responsive, and growth/development-related.

Stress-responsive elements were widely distributed, including MYB and MYC elements (associated with drought, salt, and ABA signaling), AREs (anaerobic response), LTRs (low-temperature response), and TC-rich repeats (defense signaling). The widespread presence of these elements suggests potential regulatory involvement of *CsRdDMs* in multiple abiotic stress pathways. Light-responsive elements such as the G-box and BOX4 were also frequently detected. Notably, the promoter of *CsDCL3* contained 13 G-box elements, indicating possible regulation by light signals in small RNA biogenesis. In contrast, elements associated with plant growth and development were observed in a smaller subset of *CsRdDMs*, suggesting that developmental regulation may be limited to specific members. Notably, this analysis reports only the presence of known motifs and formal overrepresentation statistics were not computed.

### 2.6. Expression Patterns of CsRdDMs in Different Tissues

**Figure 6:**
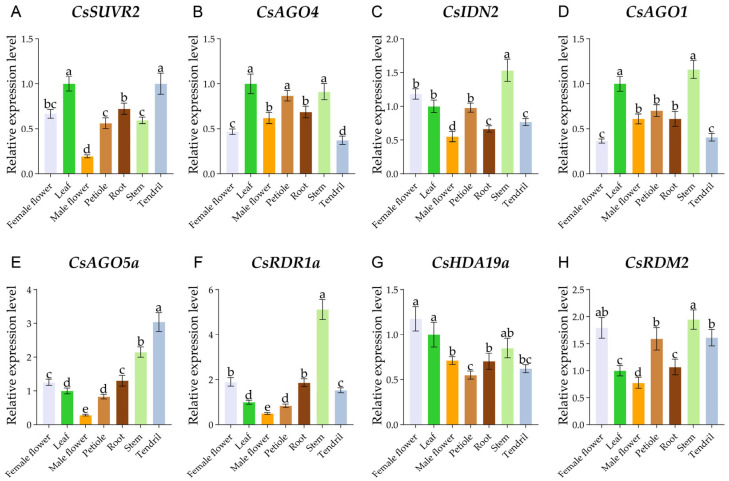
Relative expression of the eight *CsRdDMs* with the highest expression in roots in seven different tissues, including (**A**) *CsSUVR2*, (**B**) *CsAGO4*, (**C**) *CsIDN2*, (**D**) *CsAGO1*, (**E**) *CsAGO5a*, (**F**) *CsRDR1a*, (**G**) *CsHDA19a*, and (**H**) *CsRDM2*. The data presented in the graphs are expressed as the mean ± SD. Different letters denote statistically significant differences as determined by one-way ANOVA with Tukey’s post-hoc test (*p* < 0.05).

To explore the functional contributions of *CsRdDM* genes to cucumber development, their expression profiles were determined by RT-qPCR across seven distinct tissues (Figure 6). Based on the results of RT-qPCR in roots, eight representative genes were selected to analyze their unique tissue-specific expression patterns. For example, *CsAGO5a* and *CsRDM2* were significantly expressed in tendrils, while *CsIDN2*, *CsAGO1*, *CsAGO5a*, *CsRDR1a*, and *CsRDM2* were expressed at higher levels in stems. Notably, the expression of *CsRDR1a* in stems was exceptionally high, far exceeding its levels in any other tissue examined. Meanwhile, the expression of *CsRDR1a* and *CsRDM2* was relatively high in female flowers, whereas the expression of all eight genes was low in male flowers. These tissue-specific expression patterns suggest that these eight *CsRdDMs* may undergo functional differentiation and play non-redundant roles during the development of different tissues in cucumber.

### 2.7. Expression Patterns of CsRdDMs Under Abiotic Stress

**Figure 7:**
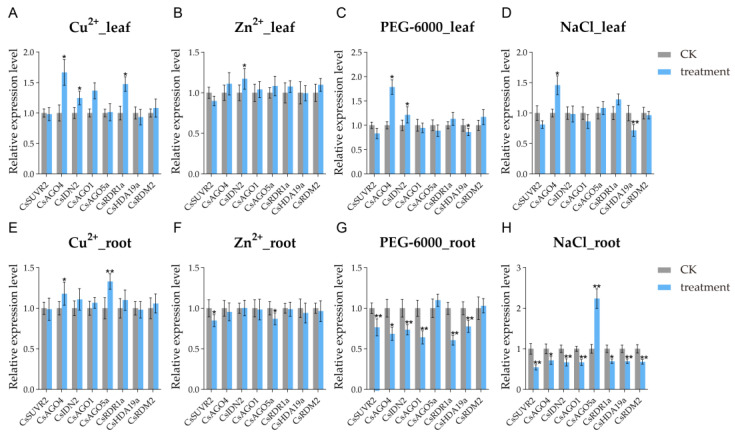
Expression analysis of *CsRdDMs* in leaf tissues under the treatment of Cu^2+^ (**A**), Zn^2+^ (**B**), PEG-6000 (**C**), and NaCl (**D**), and root tissues under the treatment of Cu^2+^ (**E**), Zn^2+^ (**F**), PEG-6000 (**G**), and NaCl (**H**) using RT-qPCR. CK (Control group) represents plants grown in standard Hoagland’s solution. The data presented in the graphs are expressed as the mean ± SD. Asterisks indicate a significant difference between the treatment and its corresponding control group for each gene, as determined by a two-tailed Student’s t-test (* *p* < 0.05, ** *p* < 0.01).

To investigate the potential functional role of the RdDM pathway genes in abiotic stress responses, the expression responses of *CsRdDM* genes were evaluated in cucumber roots and leaves under four abiotic stress conditions Cu^2+^, Zn^2+^, NaCl, and PEG-6000 treatments by RT-qPCR (Supplementary Figure S3). Notably, eight representative *CsRdDM* genes exhibited distinct patterns of stress-type and tissue-specific expression, suggesting their differential regulation in response to the environment. Under copper (Cu^2+^) stress, *CsAGO4*, *CsIDN2*, and *CsRDR1a* exhibited significant upregulation in leaves, while *CsAGO4* and *CsAGO5a* were significantly upregulated in roots. In contrast, under zinc (Zn^2+^) stress, most *CsRdDM* genes were repressed in roots (Figure 7F) but generally upregulated in leaves (Figure 7B). Although these expression changes did not meet the threshold for statistical significance, they still implied the existence of tissue-specific regulatory differences in response to Zn^2+^ stress. Under PEG-induced drought stress, the expression levels of *CsAGO4* and *CsIDN2* were significantly elevated in leaves (Figure 7C), whereas their expression in roots generally displayed a declining trend (Figure 7G). Furthermore, under NaCl treatment, *CsAGO5a* was extremely significantly upregulated in roots, while only selective induction of specific genes was detected in leaves (Figure 7D). Notably, with the exception of *CsAGO5a*, the expression of the remaining seven target genes was significantly or even extremely significantly downregulated in roots (Figure 7H). Taken together, these results demonstrate that in roots, *CsRdDM* genes tend to be upregulated under Cu^2+^ stress but are largely suppressed under Zn^2+^, PEG-6000, and NaCl treatments. This pattern implies that cucumber roots may possess a more specialized and robust RdDM-mediated response to copper stress, whereas the other three stressors might trigger adaptive responses through the downregulation of RdDM-related gene expression.

## 3. Discussion

The RdDM pathway is a fundamental epigenetic mechanism that underpins genome integrity and environmental adaptability in plants. While this pathway has been extensively characterized in *A. thaliana* [14] and economically important crops such as tomato [26] and grapevine [27], its structural and functional organization in Cucurbitaceae, particularly in cucumber, has remained poorly understood. In this study, we performed the genome-wide identification and characterization of RdDM pathway components in *C. sativus*, uncovering 67 *CsRdDM* genes and providing new insights into their evolutionary diversification, gene structure, and stress-responsive behavior.

The identification of diverse *CsRdDM* gene families, distributed unevenly across the genome and displaying structural variability, suggests an intricate evolutionary history shaped by both developmental requirements and environmental selection pressures. For instance, *CsAGO7* exhibits an atypical gene structure with a markedly reduced number of exons compared to its paralogs. Given that the PAZ and PIWI domains are essential for AGO proteins to bind 24-nt small RNAs (sRNAs), such structural variations resulting from exon loss could alter the affinity or specificity of *CsAGO7* for its RNA targets. This structural deviation parallels similar patterns observed in the AGO4/6 subfamily of *Arabidopsis*, which is known to mediate tissue-specific transposable element silencing [28]. Given that the PAZ and PIWI domains are essential for AGO proteins to bind 24-nt small RNAs (sRNAs), such structural variations resulting from exon loss could alter the affinity or specificity of *CsAGO7* for its RNA targets. This structural deviation parallels similar patterns observed in the AGO4/6 subfamily of *Arabidopsis*, which is known to mediate tissue-specific transposable element silencing. However, changes in exon number are not the only genomic feature affecting the function of *CsRdDM* family genes. As dynamically changing regulatory elements in the genome, transposable elements (TEs) and their distribution in gene regions may also profoundly influence the functional performance of these genes.

Gene duplication has also contributed significantly to the expansion and diversification of the RdDM pathway. For example, the tandem duplication of *CsRDR1a* and *CsRDR1b* on chromosome 5 might confer a dosage advantage in double-stranded RNA synthesis, thereby enhancing sRNA biogenesis under stress conditions. This mechanism has been widely reported in adaptive responses in plants [29]. The close genomic proximity of these paralogs implies potential co-regulation and functional redundancy. To further support this potential co-regulation, we examined the promoter sequences of the duplicated *CsRDR1a* and *CsRDR1b*, and identified some common cis-regulatory elements, including AAGAA-motif, ARE, MYB, ERE, Box4, GT1-motif, GATA-motif, AE-box, and circadian. The presence of these common cis-regulatory elements provides evidence for the possibility of their co-regulation, suggesting that *RDR1* duplication may have contributed to the epigenetic adaptability of cucumber.

Additional complexity arises from the promoter architecture of *CsRdDM* genes. The enrichment of TC-rich repeats, often associated with general stress-responsive elements, supports their regulatory involvement in defense mechanisms. Notably, the promoter of *CsDCL3* contains multiple G-box elements, which are recognized by bZIP and bHLH transcription factors that mediate light signaling and environmental cue integration [30]. The regulatory complexity of the *CsRdDM* pathway is further underscored by the intricate architecture of its promoter regions. Under PEG treatment, genes such as *CsAGO4* and *CsIDN2* were significantly upregulated in leaves. Given that abscisic acid (ABA) serves as a core signaling molecule in plant responses to drought stress, this expression pattern aligns with the regulatory logic of ABA-mediated stress responses, and provides more direct support at the expression level for the association between these *CsRdDM* genes and ABA-mediated stress regulation. Additionally, MYB and MYC binding motifs are widely distributed in the promoters of *CsRdDM* genes; notably, MYB and MYC family transcription factors are key downstream effectors of the ABA signaling pathway. This indicates that *CsRdDM* genes are closely integrated into the ABA regulatory network, and as a core link in abiotic stress responses, they participate in the epigenetic regulation of such responses [31]. These features suggest that *CsRdDM* genes function as molecular hubs coordinating developmental and stress-responsive signaling networks.

Expression profiling under abiotic stresses revealed that *CsRdDM* genes employ finely tuned, tissue-specific regulatory strategies. Zn^2+^ and PEG-induced drought stress elicited contrasting expression patterns between roots and leaves. In roots, a general transcriptional repression was observed, potentially serving as an energy conservation mechanism to minimize metabolic costs during adverse conditions. In contrast, leaves exhibited an upregulation of genes such as *CsAGO4*, potentially to maintain redox homeostasis and protect against oxidative damage. This spatially differentiated response aligns with observations in barley, where salt stress represses photosynthesis-related genes in roots but upregulates them in leaves to support systemic adaptation [30,31]. These organ-specific responses may be coordinated by long-distance signaling, including mobile 24-nt siRNAs, which can move through the phloem to direct DNA methylation in distal tissues [32]. Grafting experiments have confirmed that siRNAs generated in scions can travel to rootstocks and mediate gene silencing, demonstrating the systemic propagation of RdDM-mediated signals from source to sink organs [12]. This bidirectional communication supports the hypothesis that RdDM plays a central role in maintaining whole-plant epigenetic homeostasis under stress conditions.

Among all *CsRdDM* genes, *CsAGO4* stands out as a key regulator of the abiotic stress response in cucumber. Specifically, *CsAGO4* was strongly induced in leaves under three critical stresses—Cu^2+^, PEG-6000 (simulated drought), and NaCl—highlighting its core role in the adaptation of aboveground tissues to diverse abiotic stresses. This observation aligns with the function of AGO family members in stress tolerance across plant species. For instance, in apple (*Malus domestica*), *MdAGO1* contributes to reactive oxygen species (ROS) scavenging, ionic homeostasis maintenance, and enhanced polyamine accumulation under salt stress; by contrast, *MdAGO1*-RNAi lines exhibit significantly reduced photosynthetic capacity when exposed to saline conditions [33]. Meanwhile, *CsAGO5a* showed distinct tissue-specific induction patterns: it was significantly upregulated in roots under Cu^2+^ and NaCl stresses, implying a specialized role in belowground tissue responses to heavy metal and salt stress. However, we emphasize that similar expression patterns alone are insufficient to confirm conserved function; functional validation—such as analyses of mutant phenotypes—will be required to verify whether *CsAGO4* and *CsAGO5a* exert analogous roles in stress tolerance.

The potential for transgenerational epigenetic inheritance warrants further exploration. In *Arabidopsis*, Pol IV-dependent RdDM has been shown to mediate heritable DNA methylation changes in response to heat stress [16,34], offering exciting opportunities for crop improvement. Future studies could leverage targeted epigenetic editing technologies, such as CRISPR/dCas9-DRM2 fusions, to functionally validate the regulatory roles of key *CsRdDM* genes like *CsRDR1c* [35]. Additionally, exploiting the concept of “epigenetic stress memory” may offer a promising non-transgenic approach to enhance multi-stress tolerance in cucumber. Recent studies in Arabidopsis have shown that RdDM-induced methylation changes can persist in progeny and are associated with improved growth traits under elevated CO_2_ conditions [36].

Although this study establishes a foundational understanding of the RdDM pathway in cucumber, several limitations remain and highlight future research directions. The current findings are primarily based on RT-qPCR assays, which provide insights into gene expression dynamics but do not directly confirm DNA methylation patterns. Future studies should utilize targeted and comprehensive DNA methylation assays to elucidate the mechanistic links between *CsRdDM* gene activity and precise locus-specific DNA methylation. Specifically, techniques such as whole-genome bisulfite sequencing (WGBS)—the gold standard for genome-wide methylation profiling—and emerging nanopore-based direct methylation detection (which enables single-molecule resolution without bisulfite treatment) would be well-suited for this purpose. Moreover, the current experimental design assessed early transcriptional responses (4-h treatments), which do not reflect long-term or transgenerational epigenetic effects. Future investigations should therefore extend to later time points and assess the persistence and inheritance of RdDM-mediated epigenetic modifications.

## 4. Materials and Methods

### 4.1. Identification of CsRdDMs

To comprehensively identify members of the RdDM pathway in cucumber, a Hidden Markov Model (HMM) based search was conducted [37]. First, the protein sequences of known *A. thaliana* RdDM components were retrieved from The Arabidopsis Information Resource (TAIR; http://www.arabidopsis.org/ (accessed on 11 February 2025)) [38]. The conserved domains from these sequences were used to build a pathway-specific HMM profile. This profile was then employed as a query to search the cucumber proteome within the Cucurbit Genomics Database (CuGenDB; http://cucurbitgenomics.org/organism/20 (accessed on 11 February 2025)) using HMMER 3.0 [39], with an E-value cutoff of 1e-5 [40]. To ensure the accuracy of the identified candidates, all putative *CsRdDM* protein sequences were submitted to InterProScan (http://www.ebi.ac.uk/interpro/ (accessed on 12 February 2025)) for validation, confirming the presence of the expected conserved domains [41]. Reciprocal BLASTP (version 2.15.0) against *Arabidopsis thaliana* (TAIR) was conducted for each candidate to confirm orthology, with expected top hits in the corresponding RdDM families.

### 4.2. Gene Structure, Physicochemical Properties, and Chromosomal Localization of CsRdDMs

The genomic coordinates, coding sequence (CDS) lengths, and exon-intron structures for each identified *CsRdDM* gene were extracted from the GFF3 annotation file of the cucumber genome. The exon-intron organization was visualized using the Gene Structure Display Server 2.0 (GSDS; http://gsds.gao-lab.org/ (accessed on 15 February 2025)) [42]. The physicochemical properties of the corresponding *CsRdDM* proteins, including molecular weight (MW), theoretical isoelectric point (pI), and amino acid (aa) length, were computed using the ProtParam tool on the ExPASy server (http://web.expasy.org/protparam/ (accessed on 16 February 2025)) [43]. The chromosomal locations of all *CsRdDM* genes were visualized using MapChart software (version 2.32) based on their genomic position information [44].

### 4.3. Phylogenetic, Conserved Motif, and Domain Architecture Analysis

To investigate the evolutionary relationships of the *CsRdDM* proteins, homologous sequences from seven other species (*A. thaliana*, *B. hispida*, *C. lanatus*, *C. moschata*, *L. siceraria*, *C. melo*, and *S. lycopersicum*) were retrieved from the TAIR, CuGenDB, and EnsemblPlants (https://plants.ensembl.org/index.html (accessed on 11 February 2025)) databases [45]. Multiple sequence alignments were performed on the full-length protein sequences using the MUSCLE algorithm implemented in MEGA 12.0 software [46,47]. Subsequently, a Neighbor-Joining (NJ) phylogenetic tree was constructed with 1000 bootstrap replicates to ensure statistical reliability [48]. Cucumber genes were named based on their phylogenetic proximity and sequence homology to their Arabidopsis orthologs.

Conserved motifs within the *CsRdDM* protein families were identified using the MEME suite (http://meme-suite.org/tools/meme (accessed on 18 February 2025)) with default parameters [49], except for setting the maximum number of motifs to 10. The conserved domain architectures, previously identified by InterProScan, and the MEME-derived motifs were visualized together using TBtools software (version 2.148) to facilitate comparative analysis [50].

### 4.4. Cis-Regulatory Element Analysis in Promoters

To predict the regulatory networks governing the *CsRdDM* genes, the 2000 bp nucleotide sequences upstream of the translational start codon (ATG) for each gene were extracted from the cucumber genome. These promoter sequences were then submitted to the PlantCARE database (https://bioinformatics.psb.ugent.be/webtools/plantcare/html/ (accessed on 20 February 2025)) for the identification of putative cis-regulatory elements [51]. The identified elements were categorized based on their annotated functions (e.g., stress response, light response, development) for further analysis.

### 4.5. Plant Material, Growth Conditions, and Abiotic Stress Treatments

Seeds of cucumber (‘JinYan 4’) were surface-sterilized, rinsed, and germinated on moist filter paper for 24 h. The germinated seedlings were then sown in a substrate mixture of peat, vermiculite, and perlite (3:1:1, *v*/*v*/*v*). Seedlings were grown in a controlled environment chamber (Haibo, Changzhou, China) under a 16-h light (12,000 Lux, 26 °C)/8-h dark (22 °C) photoperiod.

At the two-leaf stage, seedlings were carefully transferred to a hydroponic system containing Hoagland’s complete nutrient solution. The purpose of the 24-h dark treatment was to enhance the hydroponic adaptability of plants. After the completion of the dark period, the plants were returned to standard light conditions (16/8-h light/dark cycle at 200 μmol·m^−2^·s^−1^) for 24 h, followed by stress treatment. For heavy metal stress, the solution was replaced with Hoagland’s solution supplemented with either 50 µM CuSO_4_ or 100 µM ZnSO_4_ [52]. For salinity and drought stress, solutions containing 150 mM NaCl or 10% (*w*/*w*) PEG-6000 were used [53,54], respectively. Control plants were maintained in the standard Hoagland’s solution.

Root and leaf samples were harvested 4 h after the initiation of treatment [55,56,57]. Three independent biological replicates were collected for each treatment group. Samples were immediately frozen in liquid nitrogen and stored at −80 °C until RNA extraction.

### 4.6. RT-qPCR Analysis

Total RNA was extracted from the collected samples using the SteadyPure Plant Total RNA Extraction Kit (Accurate Biotechnology, Changsha, China) according to the method provided by the manufacturer. 1 µg of total RNA was synthesized as first-strand cDNA from 1 µg of total RNA using the HiScript III 1st Strand cDNA Synthesis Kit (Vazyme, Nanjing, China).

Real-time quantitative PCR (RT-qPCR) was performed on a quantitative thermal cycler using ChamQ SYBR qPCR Master Mix (Vazyme, Nanjing, China). Eight primers specific for genes highly expressed in roots were designed using Primer 5.0 software (see Supplementary Table S3 for primer sequences). The cucumber actin gene (*CsActin*) was used as an internal reference for normalization. The relative expression levels of each target gene were calculated using the 2^−ΔΔCt^ method. Three technical replicates of all RT-qPCR reactions were performed for each biological sample. GraphPad Prism v10.4.0 software was used for statistical analysis and graph generation.

## 5. Conclusions

This study presents the first comprehensive genome-wide characterization of 67 *CsRdDM* genes in cucumber. Phylogenetic and structural analyses revealed substantial evolutionary divergence and diversification across gene families. Promoter element analysis indicated that *CsRdDM* genes are intricately integrated into both developmental and stress-responsive regulatory networks. Expression profiling under salt, drought, and heavy metal stress conditions demonstrated that these genes exhibit complex, tissue-specific regulatory patterns. Among the identified genes, *CsAGO4* emerged as a key candidate involved in mediating systemic responses to abiotic stress. Additionally, the contrasting expression patterns observed between roots and leaves suggest an antagonistic regulatory strategy, emphasizing the spatial specificity and adaptability of RdDM-mediated regulation. Collectively, these findings provide a theoretical foundation and a set of candidate genes for future functional validation. Moreover, based on the preliminary evidence presented in this study, this work not only identifies candidate epigenetic regulators for future functional studies, but also offers critical clues for subsequent investigations into epigenetic regulation of stress tolerance.

## Data Availability

Data are contained within the article and Supplementary Materials.

## Supplementary Materials

The following supporting information can be downloaded at: https://www.mdpi.com/article/10.3390/plants14182908/s1, Figure S1: Protein domain analysis of *CsRdDM* genes. Different colored boxes represent distinct patterns, and letters (A–N) denote different subfamilies; Figure S2: Gene structure analysis of *CsRdDM* genes, and letters (A–J) denote different subfamilies; Figure S3: Expression analysis of *CsRdDM* genes under different heavy metals, PEG-6000, and NaCl treatments. Data are shown as relative expression level differences between the treatment and control groups, with the difference values ranging from −1.50 to 2.00; Table S1: Physical and chemical property analysis of *CsRdDM* proteins; Table S2: Sequence homology alignment statistics of *CsRdDM* genes; Table S3: Domains and Pfam Accession of *CsRdDM* genes; Table S4: Forward and reverse primer sequences of *CsRdDM* genes.
