# Supplementary figures and images for: Genome-Wide Identification and Expression Profiling of the RNA-Directed DNA Methylation Pathway Genes in Cucumis sativus L
Source: Plants (Basel). 2025 Sep 18;14(18):2908. doi: 10.3390/plants14182908 (PMC12473810; doi:10.3390/plants14182908)

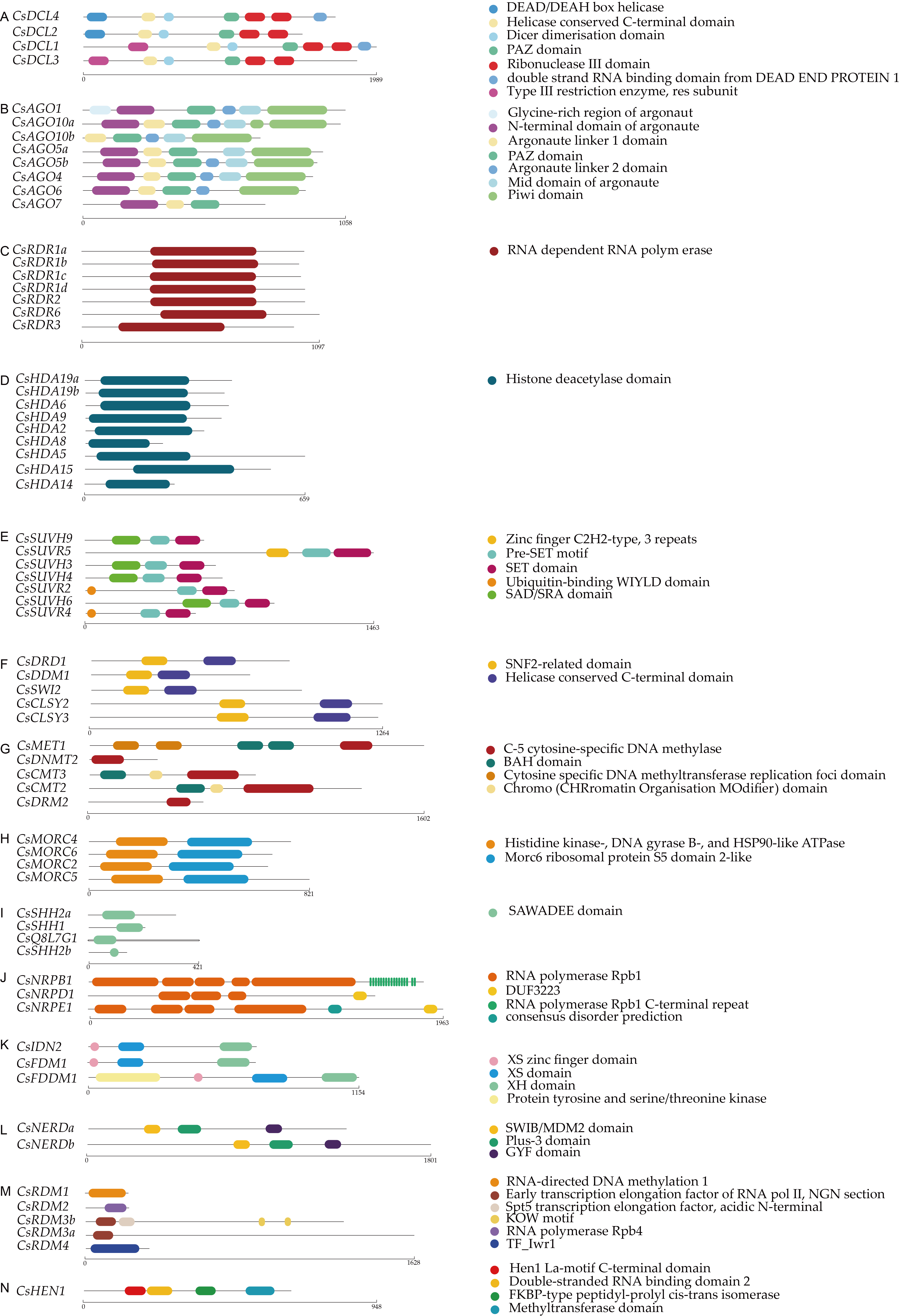

Supplement: Supplementary file 1 [file plants-14-02908-s001.zip › Fig.S1.tif]

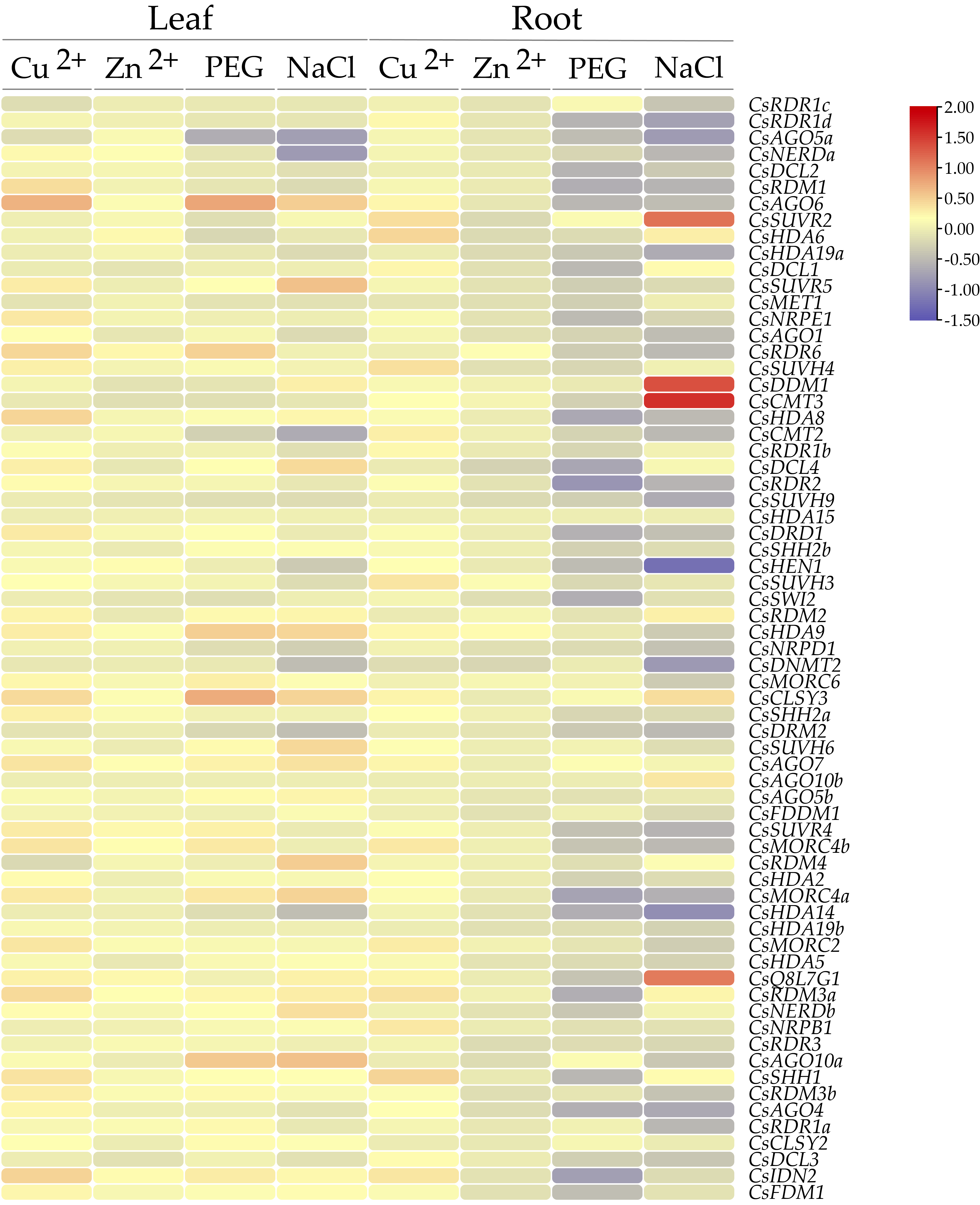

Supplement: Supplementary file 1 [file plants-14-02908-s001.zip › Fig.S3.tif]
